# Comparison of the Efficacy and Safety of Metformin-Based Combination Therapy Versus Metformin Alone in Children and Adolescents With Type 2 Diabetes Mellitus: A Meta-Analysis

**DOI:** 10.7759/cureus.35014

**Published:** 2023-02-15

**Authors:** Raza A Khan, Nidhi Patel, Atunde Folajimi, Bansari Raveena Bai, Vrushak Patel, Praveen Kumar Komminni, Sujith K Palleti, Shamsha Hirani

**Affiliations:** 1 General Medicine, The University Hospital of South Manchester NHS Foundation Trust, Manchester, GBR; 2 Internal Medicine, Gujarat Medical & Education Research Society (GMERS), Vadodara, IND; 3 Neurology, NES Healthcare, Aylesbury, GBR; 4 Internal Medicine, Peoples University of Medical and Health Sciences For Women, Nawabshah, PAK; 5 Internal Medicine, Suraksha Hospital, Khammam, IND; 6 Nephrology, Edward Hines Jr. Veterans Administration Hospital, Hines, USA; 7 Nephrology, Loyola University Medical Center, Maywood, USA; 8 Cardiology, Baqai Hospital, Karachi, PAK

**Keywords:** diabetes type 2, combination therapy, efficacy, children and adolescents, metformin

## Abstract

The aim of this meta-analysis is to synthesize and critically evaluate the available evidence on the comparison of the efficacy and safety of metformin-based combination therapy versus metformin alone in children and adolescents with type 2 diabetes mellitus (T2DM). We performed the present meta-analysis according to the Preferred Reporting Items for Systematic Reviews and Meta-analyses (PRISMA) guidelines. Eligible studies were identified using electronic searches for randomized controlled trials (RCTs) using PubMed, the Cochrane Central Register of Controlled Trials (CENTRAL), and clinicaltrial.gov from inception to 31 January 2023. The outcomes examined in this meta-analysis included change from baseline in glycated hemoglobin (HbA1C) (%), fasting plasma sugar (FPG) (mg/dl), and the number of individuals experiencing adverse events. Three studies met the criteria and were included in the meta-analysis. The reduction of HbA1C was significantly higher in metformin-based combination therapy (MD: -1.19, 95% CI: -2.05, -0.33, p-value: 0.007). No significant difference was reported between patients randomized in metformin-based combination therapy and metformin alone (MD: -18.67, 95% CI: -50.17, 12.84, p-value: 0.25). In conclusion, the present meta-analysis found that the reduction in HbA1C was significantly higher in patients receiving metformin-based combination therapy compared to metformin alone. No significant difference was found between the two groups in terms of the change in fasting plasma glucose (FPG) from the baseline. In relation to safety, no significant difference was found in the incidence of adverse events and serious adverse events between the two groups.

## Introduction and background

Type 2 diabetes mellitus (T2DM) has become a major health concern among children and adolescents in recent years [1]. The development of T2DM is influenced by factors such as genetics, family history, and lifestyle changes, including exercise and diet [1]. The incidence of T2DM in children and adolescents has increased significantly from 2007 to 2017, with an overall annual increase of 4.8% [2]. This rise in T2DM increases the risk of comorbidities such as hypertension, hyperglycemia, and dyslipidemia [3].

The management of T2DM in children and adolescents typically involves pharmaceutical interventions and lifestyle modifications. The US Food and Drug Administration (FDA) has approved four drugs to treat T2DM in this population, including insulin, exenatide, liraglutide, and metformin [4]. Metformin is often the initial medication used to control elevated blood sugar levels, particularly in overweight children and adolescents [5]. It achieves this by reducing glucose absorption in the intestines, limiting glucose production in the liver, and enhancing insulin sensitivity [6].

Using only metformin as the first treatment when diagnosed with T2DM reduces the fear of hypoglycemia and minimizes side effects compared to starting with a combination of medications [7]. However, relying solely on metformin may not consistently achieve the target HbA1c level due to the complex nature of T2DM. Additionally, the gradual approach to treatment may result in therapeutic and clinical inactivity, potentially leading to persistent high blood sugar levels and increasing the risk of long-term complications associated with T2DM [7]. On the other hand, early combination therapy can provide consistent and greater reductions of Hb1Ac owing to complementary and synergistic mechanisms of action [8]. This can be accomplished without significantly increasing the risk of hypoglycemia with the use of newer antihyperglycemic agents like GLP-1 receptor agonists (GLP-1RA), DPP-4 inhibitors (DPP-4I), and SGLT-2 inhibitors (SGLT-2I) due to their glucose-dependent mechanism of lowering blood glucose [9].

There is currently no universally agreed-upon set of guidelines for the use of combination therapy for diabetes drugs in children and adolescents. However, the International Society for Pediatric and Adolescent Diabetes (ISPAD) provides recommendations based on the latest evidence and expert consensus [10]. These recommendations generally involve starting with lifestyle modifications, followed by monotherapy with a single oral hypoglycemic agent or insulin therapy, and adding additional drugs as needed to achieve glycemic targets. The present meta-analysis aims to synthesize and critically evaluate the available evidence on the use of diabetes medications as monotherapy or metformin-based combination therapy in children and adolescents with T2DM. The purpose of this study is to provide a synthesis of the current evidence on the combination therapies used to treat T2DM in this population in order to better understand its efficacy and safety and address any gaps in the literature. A previous review focused on adult populations and showed that results for add-on therapies to metformin were similar to those for monotherapies [11]. However, no such meta-analysis has been conducted on children and adolescents. Therefore, this meta-analysis has been conducted to synthesize and critically evaluate the available evidence on the comparison of efficacy and safety of metformin-based combination therapy versus metformin alone in children and adolescents with T2DM.

## Review

Methodology

We performed the present meta-analysis according to the Preferred Reporting Items for Systematic Reviews and Meta-analyses (PRISMA) guidelines.

Eligibility Criteria

A study was eligible if it was a randomized control trial (RCT) comparing metformin-based combination therapy with metformin alone in children and adolescents with T2DM. We required that all studies have a minimum of six months follow-up. No restrictions were placed on the year of publication. We excluded studies that were observational in nature.

Search Strategy and Study Selection

Eligible studies were identified using electronic searches for RCTs using PubMed, the Cochrane Central Register of Controlled Trials (CENTRAL), and clinicaltrial.gov from inception to 31 January 2023. Key terms used to search for relevant articles included “type 2 diabetes mellitus”, “metformin”, and “antidiabetic drugs”. A systematic search strategy was employed utilizing mesh terms and boolean operators to identify relevant articles in the literature. References lists of all included articles were also manually searched. The search was performed by two authors independently.

Two investigators analyzed records retrieved from online databases and clinical registries. After removing duplicates, studies were screened based on abstract and title initially. Full texts of eligible records were obtained and assessed for predefined inclusion and exclusion criteria. All records identified in searches were stored in EndNote X9 (Thomson Reuters, New York). Any disagreement between the two authors in the search and study selection process was resolved via consensus.

Data Extraction and Outcomes Measure

All data were independently extracted by two authors into standardized electronic forms. Data extracted included author name, year of publication, groups, sample size, type of drug, follow-up duration, and participants’ characteristics. Outcomes examined in this meta-analysis included change from baseline in glycated hemoglobin (HbA1c) (%), fasting plasma sugar (FPG) (mg/dl), and the number of individuals experiencing adverse events and serious adverse events. All disagreements between the two authors were resolved by consensus.

Quality Assessment

The Cochrane Collaborations Risk of Bias tool was used to assess the quality of the included RCTs. All six domains in the risk of bias tools were assessed, including “random sequence generation”, “allocation concealment”, “blinding”, “selective outcome reporting”, “incomplete outcome data”, and “other bias”. Each domain was categorized as having a low, high, or unclear risk of bias. Quality assessment was performed by two authors independently, and any disagreement between the two authors was resolved by consensus.

Data Analysis

The data analysis was performed using RevMan version 5.4.1 (Review Manager, The Cochrane Collaboration, London, United Kingdom). Continuous outcomes, such as changes in HbA1C and fasting plasma glucose (FPG) from baseline, were analyzed using mean difference (MD) and 95% confidence intervals. Dichotomous variables, such as adverse events and serious adverse events, were analyzed using risk ratio (RR) with 95% confidence intervals, along with the number of patients who experienced adverse events compared to the total number of participants in each study. A cut-off of statistical significance was set at a p-value of less than 0.05. Heterogeneity among the study results was assessed using I-square, with a random effect model applied when the I-square value was greater than 50%; otherwise, the fixed-effect model was used. 

Results

**Figure 1:**
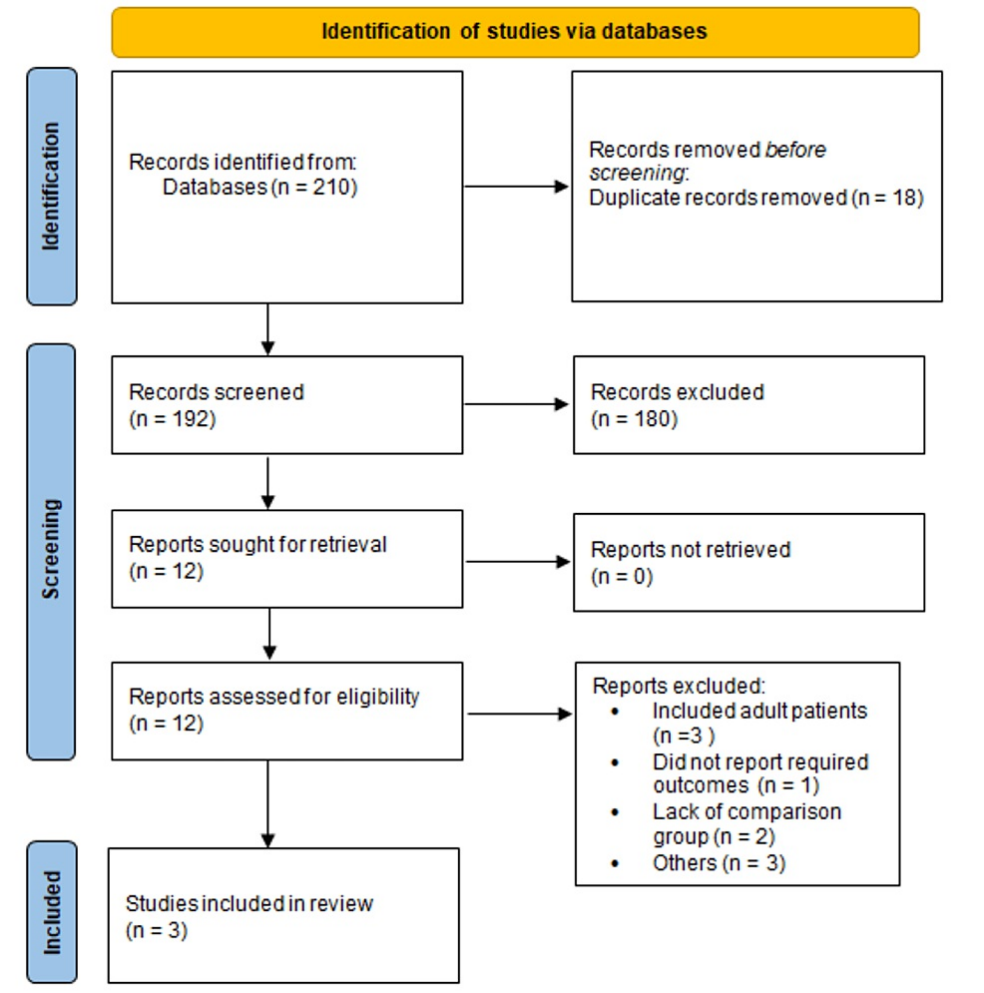
PRISMA flowchart of selection of studies PRISMA: Preferred Reporting Items for Systematic Reviews and Meta-Analyses

**Figure 2:**
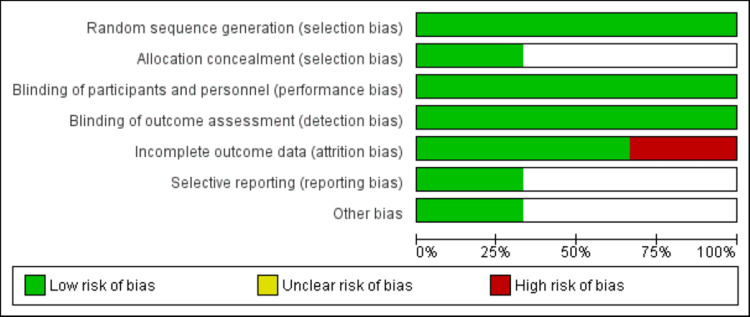
Risk of bias graph

Figure 1 shows the process of study selection. Overall online searching yielded 210 papers. After removing duplicates, 192 articles were screened using the titles and abstracts of studies. The full text of 12 articles was retrieved and assessed for the inclusion and exclusion criteria. Finally, three studies met the criteria and were included in the meta-analysis [12-14]. Out of these three studies, two were published [12,14] and one was an unpublished clinical trial [13]. The characteristics of the included studies are presented in Table 1. The sample size of the studies ranged from six to 220, and the majority of the participants were female. Figure 2 shows the risk of bias graph.

**Table 1:** Characteristics of the included studies NR: Not reported

Author Name	Year	Groups	Drugs	Sample Size	Follow-up	Mean age (Years)	Females (%)
Jalaludin et al [12]	2021	Combination	sitagliptin+metformin	107	52 Weeks	14.5	65.91
		Monotherapy	metformin	113			
NCT01434186 [13]	2017	Combination	saxagliptin+metformin	4	52 Weeks	NR	NR
		Monotherapy	metformin	2			
Tamborlane et al [14]	2019	Combination	liraglutide+metformin	66	52 Weeks	14.6	61.9
		Monotherapy	Metformin	68			

Change in HbA1c From Baseline

**Figure 3:**
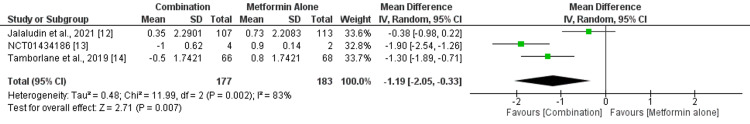
Comparison of change in HbA1C from baseline between two study groups HbA1C: glycated hemoglobin Sources: References [12-14]

The change of HbA1c from baseline was analyzed in 360 patients in three studies. In the random effect model, compared to metformin alone, the reduction in HbA1C was significantly greater in metformin-based combination therapy (MD: -1.19, 95% CI: -2.05, -0.33, p-value: 0.007) as shown in Figure 3. High heterogeneity was reported among the study results (I-square: 83%).

Change in FPG From Baseline

**Figure 4:**
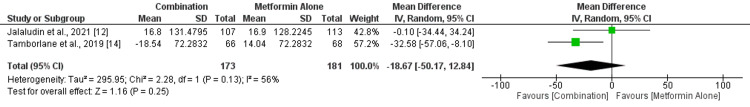
Comparison of change in FPG from baseline between two study groups FPG: fasting plasma sugar Sources: References [12,14]

The change of FPG from baseline was analyzed in 354 patients in two studies. In the random effect model, no significant difference was reported between patients randomized in metformin-based combination therapy and metformin alone (MD: -18.67, 95% CI: -50.17, 12.84, p-value: 0.25) as shown in Figure 4. High heterogeneity was reported among the study results (I-square: 56%).

Safety Analysis

Table 2 shows a comparison of adverse events and serious adverse events between patients on metformin-based combination therapy and metformin alone. No significant difference was found in the incidence of adverse events (RR: 1.04, 95% CI: 0.94, 1.15) and serious adverse events (RR: 1.72, 95% CI: 0.73-4.03) between the two groups. 

**Table 2:** Comparison of safety events RR: risk ratio; CI: confidence interval; SAE: serious adverse events

Outcome	RR (95% CI)	P-value	I-square
Adverse Events	1.04 (0.94-1.15)	0.42	0%
SAE	1.72 (0.73-4.03)	0.21	0%

Discussion

The findings of the present meta-analysis showed that patients receiving metformin-based combination therapy had greater improvement in glycemic control, as measured by HbA1C, compared to those receiving metformin alone. The meta-analysis included three randomized controlled trials (RCTs), including one unpublished study [13], which did not provide specific baseline data such as baseline BMI, baseline HbA1C, and age.

Metformin combined with exercise and diet has been the first-line treatment for type 2 diabetes mellitus (T2DM) in children and adolescents. Recently, the Food and Drug Administration (FDA) approved exenatide and liraglutide for T2DM [15]. Few studies have compared the efficacy and safety of different drugs in children and adolescents with T2DM [16-17]. Metformin has been proven to be effective and safe for treating T2DM in pediatric patients [16]. The most common adverse events associated with metformin therapy are gastrointestinal disorders such as diarrhea and abdominal pain [18].

Liraglutide has been found to be a useful addition to the available treatments for T2DM, effectively lowering blood sugar levels while preventing weight gain and hypoglycemia [19-20]. A study by Gottschalk et al. found that glimepiride reduced HbA1C similarly to metformin but resulted in greater weight gain [21]. Another study found that 52% of children failed to achieve control with monotherapy, and adding rosiglitazone to metformin improved glycemic control [22]. The present meta-analysis suggests that the addition of other hypoglycemic drugs to metformin can result in better glycemic control compared to patients receiving metformin alone.

Studies conducted in adults found that liraglutide combined with metformin has shown greater reductions in HbA1C levels compared to metformin alone [23]. Similar findings were reported in a study by Nauck et al. involving glimepiride [24]. The addition of other antidiabetic agents to metformin has been shown to enhance its glucose-lowering effects through complementary actions on different metabolic pathways. For example, metformin reduces hepatic glucose output [25] while drugs such as liraglutide or rosiglitazone target peripheral insulin resistance [22,23]. Combining these different mechanisms may provide more comprehensive and effective glucose control.

These findings are consistent with current guidelines for managing T2DM in children and adolescents. The American Diabetes Association recommends the use of metformin as first-line therapy, with the addition of other antidiabetic agents as needed to achieve glycemic goals [26]. While metformin-based combination therapy may result in greater glycemic improvement, it is important to consider potential risks and side effects associated with using multiple medications. Close monitoring and adjusting treatment regimens as necessary are important to ensure optimal glycemic control while minimizing potential harm.

Study Limitations

Certain limitations should be acknowledged. First, there were only three studies included in this meta-analysis. One of them is an unpublished study involving six patients in total. To make sure of sufficient statistical power, we did not exclude this study from the primary analysis. All three studies used different drugs with metformin and due to the limited number of studies, we could not perform a subgroup analysis. Even though in the present meta-analysis, a metformin-based combination drug was effective in reducing Hb1AC levels in individuals, the efficacy and safety of different drug combinations for T2DM in children and adolescents still require further exploration.

## Conclusions

In conclusion, the present meta-analysis found that reduction in HbA1C was significantly greater in patients receiving metformin-based combination therapy compared to metformin alone. No significant difference was found between the two groups in terms of change in FPG from baseline. In relation to safety, no significant difference was found in the incidence of adverse events and serious adverse events between the two groups. The findings of this meta-analysis need to be interpreted with caution, as the study was limited to a small number of studies. Further research is needed to establish the optimal combination of antidiabetic drugs for this population. In particular, large, randomized controlled trials that compare the different metformin-based combination therapies would help better understand the benefits and risks associated with each combination. Additionally, long-term follow-up studies are needed to assess the sustainability of glycemic control with different drug combinations, as well as the potential for long-term adverse effects. It is also important to consider the use of personalized medicine approaches, where individual patient characteristics, such as age, weight, and baseline glucose levels, are taken into account to determine the most appropriate treatment regimen.
